# Supplementary material for: Calcium-activated 14-3-3 proteins as a molecular switch in salt stress tolerance
Source: Nat Commun. 2019 Mar 13;10:1199. doi: 10.1038/s41467-019-09181-2 (PMC6416337; doi:10.1038/s41467-019-09181-2)
Supplement: Supplementary file 1 — Supplementary Information File [file 41467_2019_9181_MOESM1_ESM.pdf]

Supplementary Information

a

Bait	PKS5 peptide		
	Mascot score	Matched queries	Matched peptides
SOS2	363	14	9
Empty		ND	ND

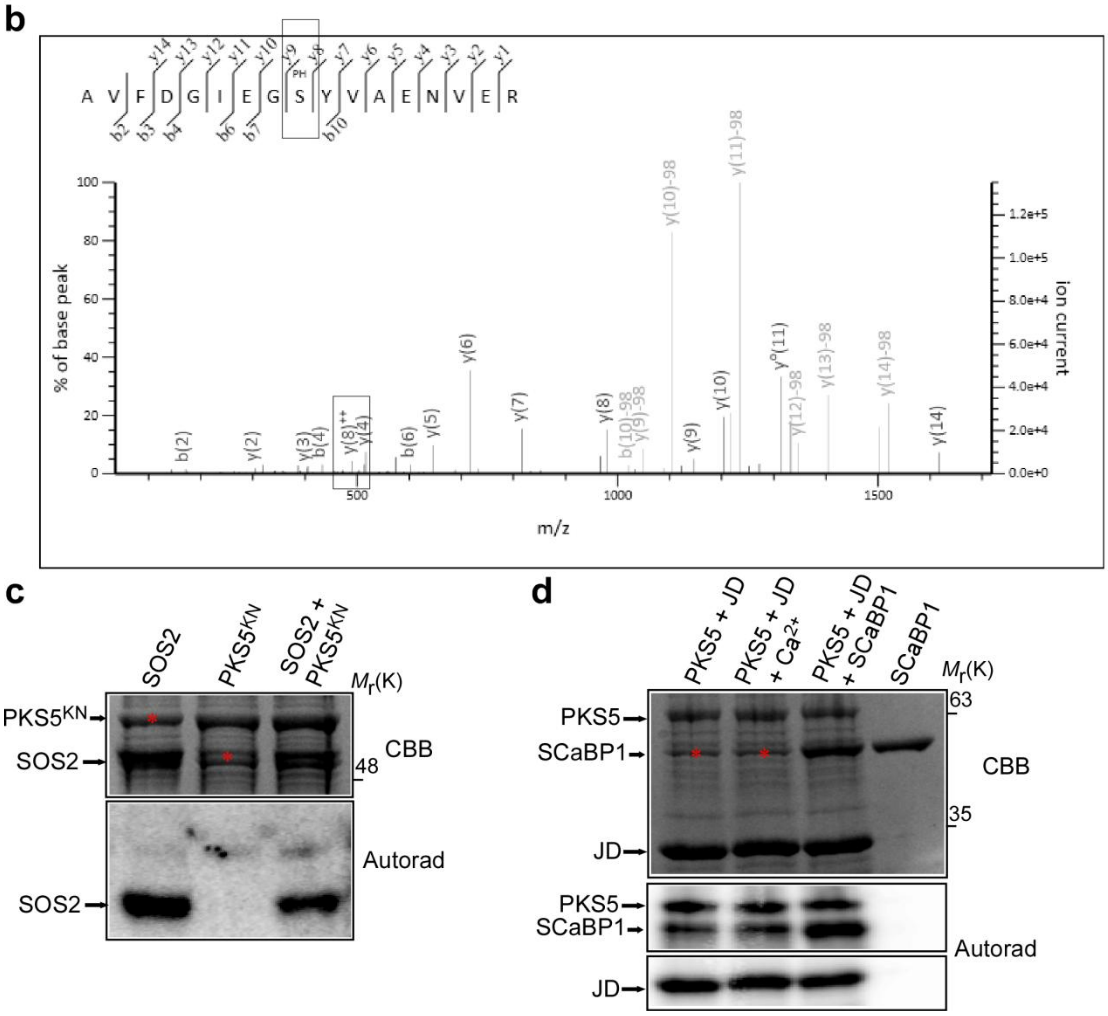

Supplementary Fig. 1 PKS5 interacts with SOS2 and phosphorylates SOS2^{Ser294} site.

(a) Arabidopsis transgenic plants Myc-SOS2 in Col-0 and Col-0 (Empty, negative control) were used in this experiment. After the immunoprecipitation with anti-Myc antibody, the proteins were subject to LC-MS/MS analysis. ND, no detected.

(b) MS spectrum shows that the peptide AVFDGIEGSYVAENVER contains phosphoserine at Ser^{294} in SOS2 after incubation with recombinant PKS5 in kinase buffer with ATP at 30°C for 30 min.

(c) *In vitro* kinase assay showing that SOS2 does not phosphorylate $PKS5^{K50N}$ (a kinase-dead type mutant). $PKS5^{KN}$, His-tagged $PKS5^{K50N}$. Asterisk indicates the non-specific band.

(d) *In vitro* kinase assay showing that 10 μM Ca^{2+} and SCaBP1 have no obvious effect on the phosphorylation of SOS2-JD by PKS5. Asterisk indicates the non-specific band.

CCB, Coomassie Brilliant Blue; Autorad, autoradiograph. The underlying Source data of c and d are provided in the Source Data file

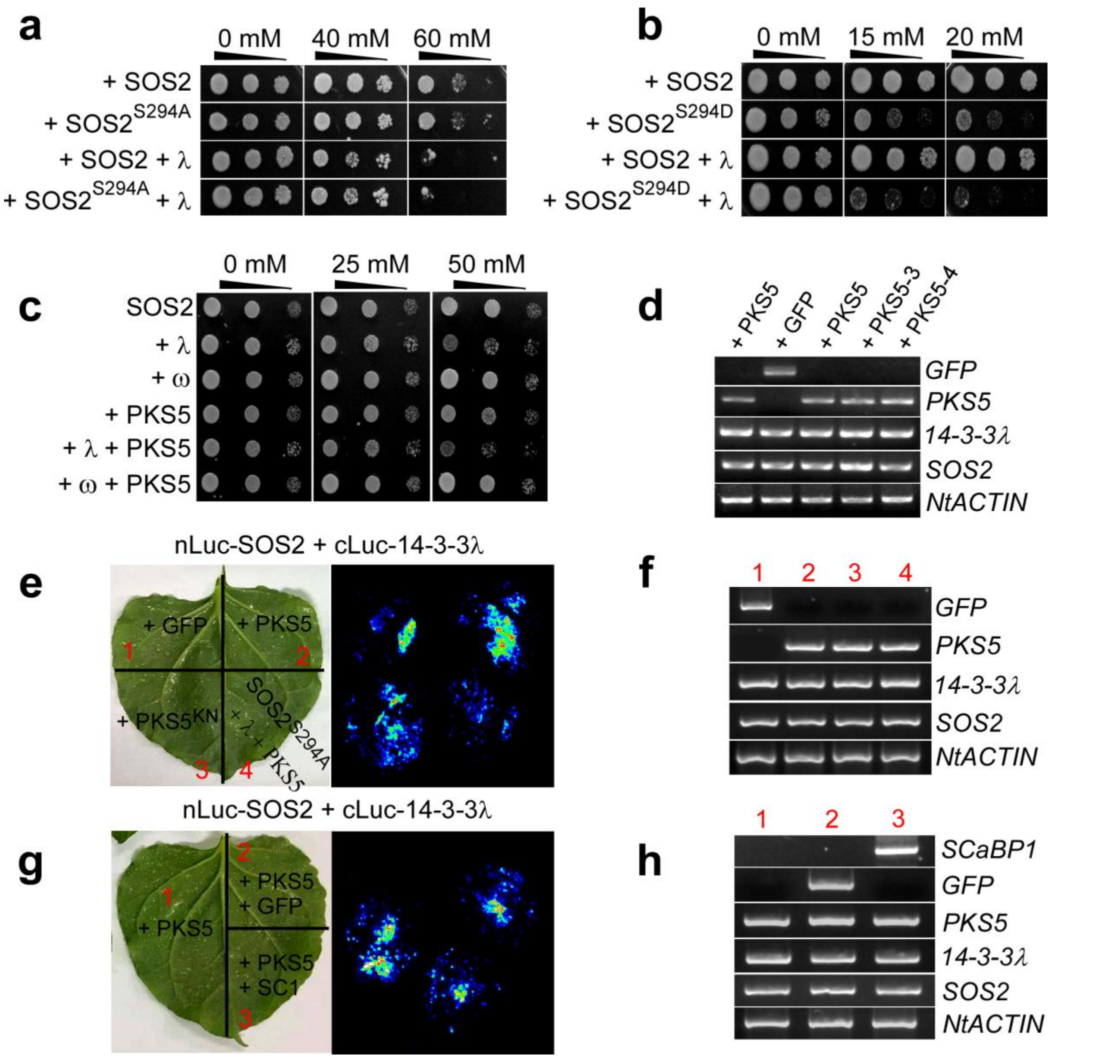

Supplementary Fig. 2 Effects of 14-3-3 proteins on phospho-dead and phospho-mimic mutations of SOS2 function in SOS recruitment system.

(a) The repression of SOS2 activity by 14-3-3λ was similar to that of SOS2^{S294A} in yeast.

(b) SOS2 Ser294-to-Asp mutation enhanced the repression of SOS2 activity by 14-3-3λ in yeast.

(c) PKS5 could not increase 14-3-3λ–mediated repression of SOS2 activity in yeast. 14-3-3ω was used as a negative control.

(d) Transient expression of indicated genes in *N. benthamiana* leaves related to Fig. 2 (c).

(e) Luciferase complementation imaging assay showing that co-expression of PKS5^{K50N} in *N. benthamiana* has no effect on the PKS5–enhanced interaction between SOS2 and 14-3-3λ, nLuc-SOS2^{S294A} served as a control.

(f) Transient expression of indicated genes in *N. benthamiana* leaves related to Supplementary Fig. 2 (e).

(g) Luciferase complementation imaging assays showing that SCaBP1 does not affect the PKS5-mediated interaction between 14-3-3λ and SOS2. SC1, SCaBP1.

(h) Transient expression of indicated genes in *N. benthamiana* leaves related to Supplementary Fig. 2 (g).

The data in (a-c), yeast cells expressing Arabidopsis *SOS1* were cotransformed with the indicated plasmids, two positive clones with 3.5 μL of serial five-fold dilutions were grown on AP medium with indicated concentration of NaCl to analyze the salt tolerance of yeast. SOS2, SOS2$^{T168D/\Delta308}$; SOS2^{S294A}, SOS2$^{T168D/\Delta308/S294A}$; SOS2^{S294D}, SOS2$^{T168D/\Delta308/S294D}$.

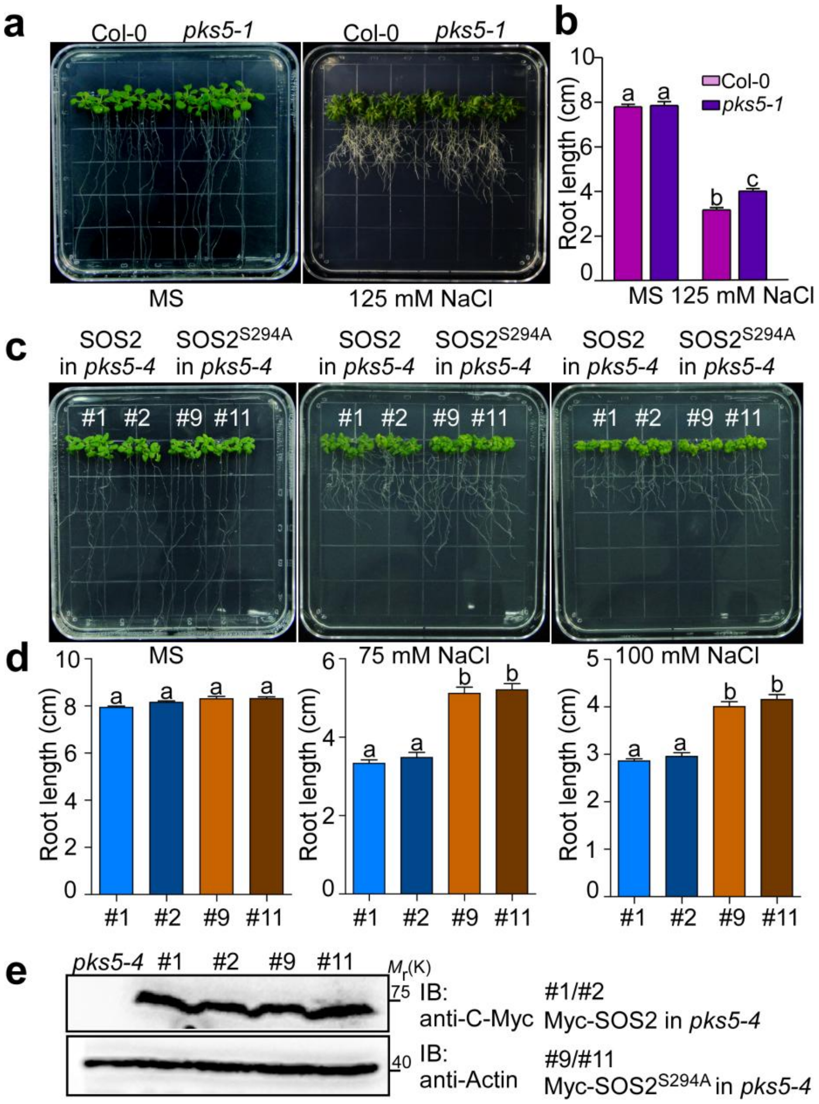

Supplementary Fig. 3 Overexpression of SOS2^{S294A} rescues the salt sensitive phenotype of *pks5-4* in Arabidopsis.

(a) Analysis of the salt phenotype of Col-0 and *pks5-1*. Five-day-old seedlings were transferred from MS to medium with 125 mM NaCl. Photographs were taken 10 days for MS and 3 weeks for media with 125 mM NaCl after transfer.

(b) Root length analysis for seedlings in (a). Error bars represent SD; $p \leq 0.05$, Student's t test; n = 15; significant difference was indicated by different lowercase letters.

(c) Analysis of the salt phenotype of two different transgenic lines of *Pro35S:6×Myc-SOS2* (#1 and #2) and *Pro35S:6×Myc-SOS2S294A* (#9 and

#11) in *pks5-4* background. Five-day-old seedlings were transferred from MS to medium with 75 or 100 mM NaCl. Photographs were taken 10 days after transfer.

(d) Root length analysis for seedlings in (c). Error bars represent SD; $p \leq 0.05$, Student’s t test; n = 12; significant difference was indicated by different lowercase letters.

(e) Protein levels of SOS2 and $SOS2^{S294A}$ transgenic plants used in (c). Actin was used as a loading control. IB, immunoblot.

Source data of b and d are provided in the Source Data file

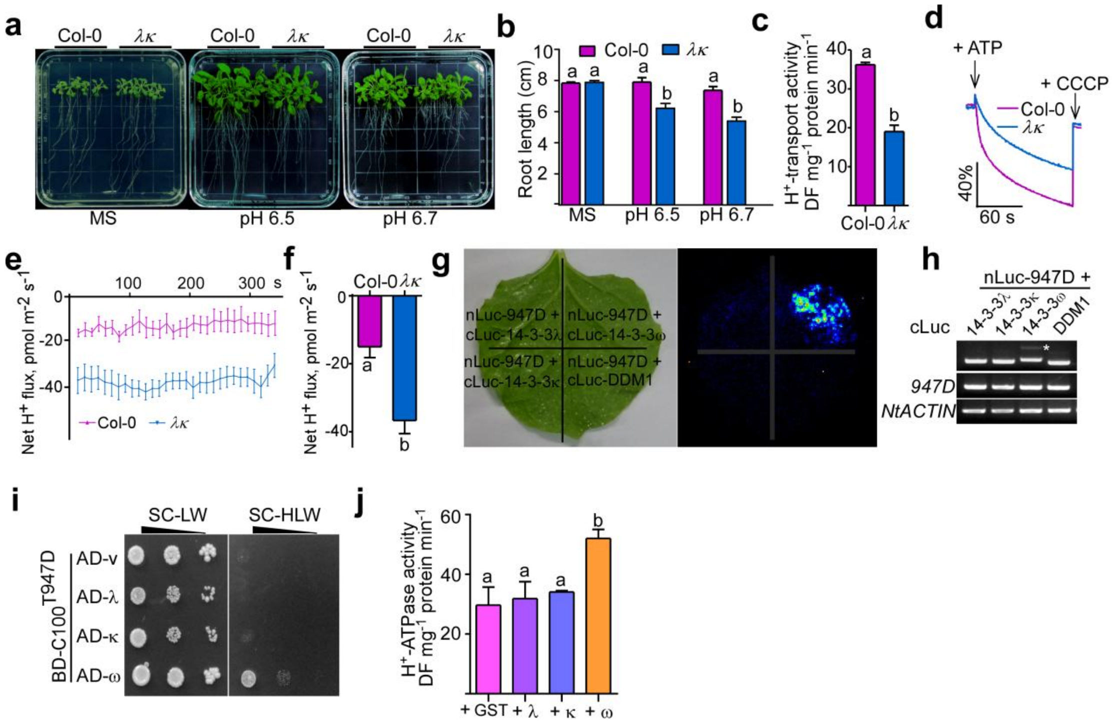

Supplementary Fig. 4 14-3-3λ and κ are critical for alkaline tolerance in Arabidopsis.

(a) Alkaline sensitive analysis of Col-0 and *14-3-3λκ* double mutant. Five-day-old seedlings were transferred from MS to medium with pH 6.5 or 6.7 adjusted by $NaHCO_3$. Photographs were taken 10 days for MS and 3 weeks for MS with high pH after transfer.

(b) Root length analysis for seedlings in (a). Error bars represent SD; $p \leq 0.05$, Student's t test; n = 15; significant difference was indicated by different lowercase letters.

(c) Comparison of PM H^+-ATPase activity in Col-0 and *14-3-3λκ* double mutant. Error bars represent SD; $p \leq 0.05$, Student's t test; n = 3; significant difference was indicated by different lowercase letters.

(d) Timely varying curves of PM H^+-ATPase activity in Col-0 and *14-3-3λκ* double mutant.

(e) The net H^+ flux analysis of Col-0 and *14-3-3λκ* double mutant by NMT. Seven-day-old seedlings were incubated in measuring solution for 10 min. Error bars represent SD; $p \leq 0.05$, Student's t test; n = 6.

(f) Calculated net H^+ fluxes from (e).

(g) 14-3-3ω interacts with AHA2 $C100^{T947D}$ but not 14-3-3λ and κ in Luciferase complementation assay. DDM1 was used as a negative control. 947D, AHA2 $C100^{T947D}$.

(h) Transient expression of indicated genes in *N. benthamiana* leaves in (g). Asterisks represent non-specific bands.

(i) Yeast two-hybrid assay shows that 14-3-3ω interacts with AHA2 $C100^{T947D}$ but not 14-3-3λ and κ. $C100^{T947D}$, AHA2 $C100^{T947D}$.

(j) Effect of recombinant 14-3-3 proteins on PM H^+-ATPase activity. Plasma membrane vesicles isolated from Col-0 were used for H^+-ATPase activity measurement when added of 500 ng/mL 14-3-3 proteins. Error bars represent SD; $p \leq 0.05$, Student's t test; n = 3; significant difference was indicated by different lowercase letters.

Source data of b, c, e and f are provided in the Source Data file

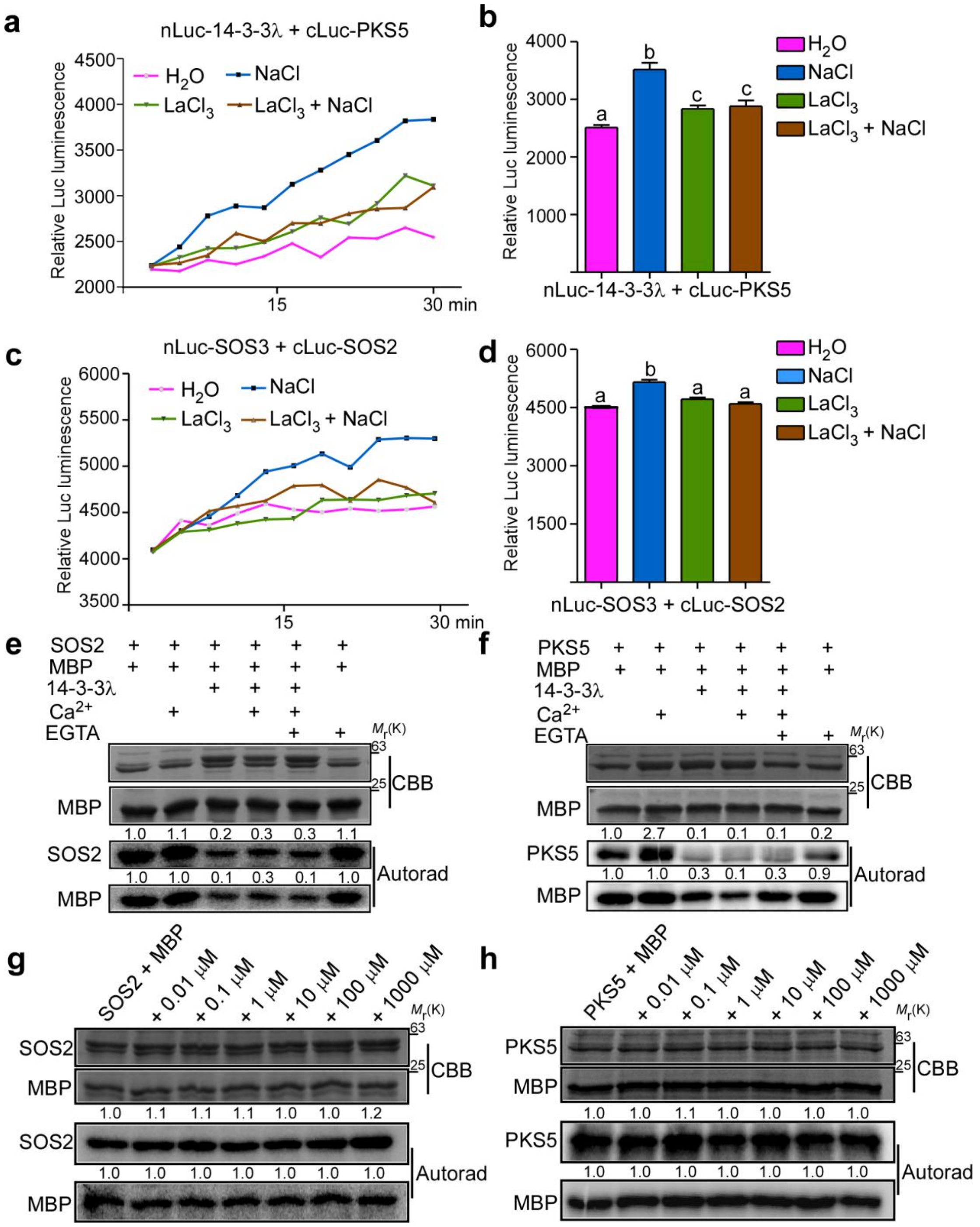

Supplementary Fig. 5 Ca^{2+} ions positively and negatively regulate SOS2 and PKS5 activities by interacting with 14-3-3 proteins, respectively.

(a) Luciferase complementation assays show that NaCl induced elevations of Ca2+ ions enhance the 14-3-3λ–PKS5 interactions. Infiltrated leaf discs are pretreated with 50 μM of LaCl3 for 20 min, and then incubated with 1 mM of

luciferin containing 200 mM NaCl in a 96-well plate for 5-30 min. Data shown are representative of three independent experiments.

(b) Quantification of the interaction changes by different treatments shown in (a). Error bars represent SD; $p \leqslant 0.05$, Student's t test; n = 3; significant difference was indicated by different lowercase letters.

(c) Luciferase complementation assays show that NaCl induced elevations of Ca2+ ions enhance the SOS3–SOS2 interactions. Infiltrated leaf discs are pretreated with 50 μM of LaCl3 for 20 min, and then incubated with 1 mM of luciferin containing 200 mM NaCl in a 96-well plate for 5-30 min. Data shown are representative of three independent experiments.

(d) Quantification of the interaction changes by different treatments shown in (c). Error bars represent SD; $p \leqslant 0.05$, Student's t test; n = 3; significant difference was indicated by different lowercase letters.

(e and f) The addition of EGTA can rescue the Ca2+ influence on the 14-3-3λ–mediated SOS2 (e) and PKS5 (f) activities. About 1.0 μg of 14-3-3λ protein was incubated with or without the final concentration of 500 μM of CaCl2 and 5 mM of EGTA at room temperature for 30 min before kinase assay.

(g and h) In vitro kinase assays analyze the effect of Ca2+ on the SOS2 (g) and

PKS5 (h) activities. About 1.0 μg of SOS2 or PKS5 protein was incubated with the indicated concentration of CaCl2 at room temperature for 30 min before kinase assay.

Source data are provided as a Source Data file

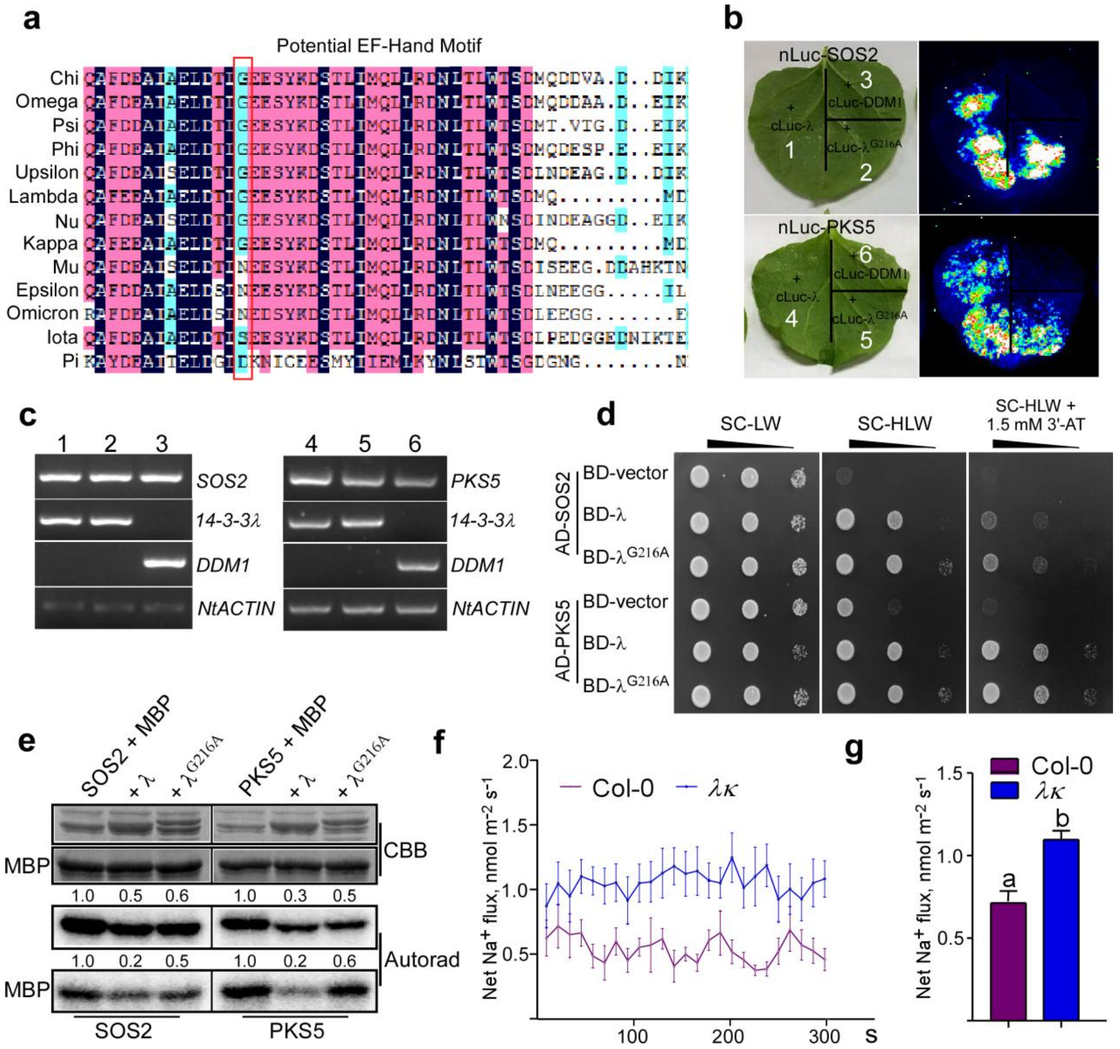

Supplementary Fig. 6 Interaction analysis of 14-3-3λ^{G216A} between SOS2 or PKS5, respectively.

(a) Alignment of other 14-3-3 proteins with 14-3-3ω at potential EF hand motif (14-3-3ω，amino acids 200–247).

(b) Luciferase complementation assays show that 14-3-3λ^{G216A} interacts with SOS2 and PKS5.

(c) Transient expression of indicated genes in *N. benthamiana* leaves in (b).

(d) Yeast two-hybrid assays show that 14-3-3λ^{G216A} interacts with SOS2 and PKS5.

(e) *In vitro* kinase assays show that the inhibition of SOS2 and PKS5 activities by 14-3-3λ^{G216A} is slightly less compared with wild type.

(f) The net Na^+ flux analysis of Col-0 and *14-3-3λκ* double mutant by NMT. Five-day-old seedlings were pretreated with 100 mM NaCl for 24 h. Error bars represent SD; $p \leq 0.05$, Student's t test; n = 7.

(g) Calculated net Na^+ fluxes from (f).

Source data of e, f and g are provided in the Source Data file

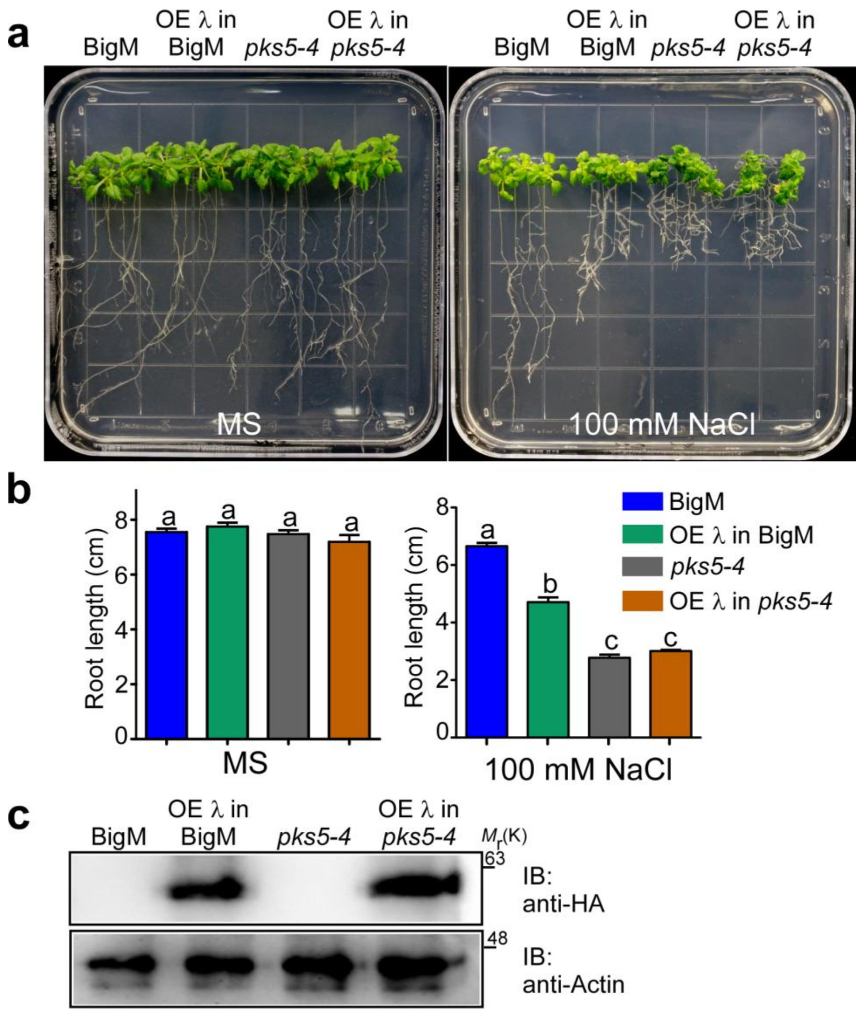

Supplementary Fig. 7 14-3-3 proteins negatively regulate the salt tolerance of Arabidopsis.

(a) Salt sensitive analysis of *pks5-4*, BigM and the transgenic plants expressing HA-14-3-3λ in the *pks5-4* and BigM background. OE λ in BigM, the *Pro35S:Flag-HA-14-3-3λ* transgenic plants in the BigM background. OE λ in *pks5-4*, the *Pro35S:Flag-HA-14-3-3λ* transgenic plants in the *pks5-4* background. Five-day-old seedlings were transferred from MS to medium containing 100 mM NaCl. Photographs were taken 10 days after transfer.

(b) Root length analysis for seedlings in (a). Error bars represent SD; $p \leq 0.05$, Student's t test; n = 12; significant difference was indicated by different

lowercase letters.

(c) Protein levels of 14-3-3λ transgenic plants used in (a). Actin was used as a loading control. IB, immunoblot.

Source data of b are provided in the Source Data file

Supplementary Table 1. Oligos used in this study

Primer	Sequence (5'-3')
**Primers for cloning**	
BD-PKS5-F	CGGAATTCATGCCAGAGATCGAGA
BD-PKS5-R	GCGTCGACAATAGCCGCGTTTGTT
BD-PKS5N-F	CGGAATTCATGCCAGAGATCGAGA
BD-PKS5N-R	GCGTCGACTCCTCTAACAAACCAA
BD-PKS5JD-F	CGGAATTCGGTTTTAAACAGATCA
BD-PKS5JD-R	GCGTCGACACTCTTCACAGCTTCT
BD-PKS5C-F	CGGAATTCTTAAACGCGTTTGATT
BD-PKS5C-R	GCGTCGACAATAGCCGCGTTTGTT
BD-AHA2 C100^{T947D}-F	CGGAATTCTGGCTCAACTTGTTTG
BD-AHA2 C100^{T947D}-R	GCGTCGACCACATCGTAGTGACTG
AD-14-3-3κ-F	CGGAATTCTGGCTCAACTTGTTTG
AD-14-3-3κ-R	GCGTCGACCACATCGTAGTGACTG
AD-14-3-3ω-F	CGGAATTCATGGCGTCTGGGCGTG
AD-14-3-3ω-R	CGGGATCCTCACTGCTGTTCCTCG
GST-14-3-3κ-F	CGGGATCCATGGCGACGACCTTAA
GST-14-3-3κ-R	GCGTCGACTCAGGCCTCATCCATC
GST-14-3-3ω-F	CGGGATCCATGGCGTCTGGGCGTG
GST-14-3-3ω-R	GCGTCGACCTGCTGTTCCTCGGTC
GST-SOS2-JD^{S294A}-F	CGGGATCCTATGTGCCTATACGAG
GST-SOS2-JD^{S294A}-R	GCGTCGACCAGGGGCCCTTCATCA
YFP^{N}-PKS5-F	CGGGATCCATGCCAGAGATCGAGA
YFP^{N}-PKS5-R	GGGGTACCAATAGCCGCGTTTGTT
YFP^{C}-SOS2-F	GCGTCGACATGACAAAGAAAATGA
YFP^{C}-SOS2-R	GGGGTACCAAACGTGATTGTTCTG
nLuc-PKS5-F	GGGGTACCATGCCAGAGATCGAGA
nLuc-PKS5-R	GCGTCGACAATAGCCGCGTTTGTT
nLuc-947D-F	GGGGTACCTGGCTCAACTTGTTTG
nLuc-947D-R	GCGTCGACCACATCGTAGTGACTG
cLuc-14-3-3κ-F	GGGGTACCATGGCGACGACCTTAA
cLuc-14-3-3κ-R	GCGTCGACTCAGGCCTCATCCATC
cLuc-14-3-3ω-F	GGGGTACCATGGCGTCTGGGCGTG

cLuc-14-3-3ω-R	GCGTCGACTCACTGCTGTTCCTCG
p414GPD-PKS5/PKS5-3/PKS5-4-F	CGGGATCCATGCCAGAGATCGAGA
p414GPD-PKS5/PKS5-3/PKS5-4-R	GCGTCGACAATAGCCGCGTTTGTT
p415GPD-14-3-3λ-F	CGGGATCCATGGCGGCGACATTAG
p415GPD-14-3-3λ-R	GCGTCGACGGCCTCGTCCATCTGC
p415GPD-14-3-3ω-F	CGGGATCCATGGCGTCTGGGCGTG
p415GPD-14-3-3ω-R	GCGTCGACCTGCTGTTCCTCGGTC
p415GPD-T/DSOS2/308S294A-R	GCGTCGACCAGGGGCCCTTCATCATTTCTCTCTACATTCTCCGCTACATAAGCGCCCTC
p415GPD-T/DSOS2/308S294D-R	GCGTCGACCAGGGGCCCTTCATCATTTCTCTCTACATTCTCCGCTACATAATCGCCCTC
14-3-3λ^{G216A}-F	GAGCTTGACACTCTGGCAGAGGAA
14-3-3λ^{G216A}-R	GTCTTTGTAGGATTCCTCTGCCAG
**Primers for RT-PCR**	
GFP-F	CGTCCAGTGCTTCTCCCGCTAC
GFP-F	CTTGATGCCGTTCTTCTGCTTG
DDM1-F	GAGGTTGCCATGAATGATGCTAAA
DDM1-R	CTTCTTCCTTGGTTGCTTCGTTTT
NtActin-F	CCACACAGGTGTGATGGTTG
NtActin-R	GTGGCTAACACCATCACCAG
PKS5-F	GAAGGTGCTAAAGTTGATGTATGGTCT
PKS5-R	CGTCATCGTGGAACTTGATCTGTTT
SOS2-F	AAAATGAGAAGAGTGGGCAAGTACG
SOS2-R	CCTCCTGTCACAAACTCCAAAACTA
14-3-3λ-F	AGGAAAACTGCTGCTGAAGATACCA
14-3-3λ-R	CCTCGTCCATCTGCTCCTGC
14-3-3κ-F	TCCGATCAGGCTTGGTTTGG
14-3-3κ-R	CAATATGCGAGTTTCTGATGATGC
14-3-3ω-F	ATGGCGTCTGGGCGTGAAG
14-3-3ω-F	GCAGCGGCAGGGATGAGTCTAG
SOS3-F	TCGAGTTTGGTGAATTTGTCCG
SOS3-R	CTGCTTGCACGAAAGCCTTATC
SCaBP1-F	ATGTCGCAGTGCGTTGACGG
SCaBP1-R	TCAGGTATCTTCAACCTGAG
