# Supplementary material for: Calcium-activated 14-3-3 proteins as a molecular switch in salt stress tolerance
Source: Nat Commun. 2019 Mar 13;10:1199. doi: 10.1038/s41467-019-09181-2 (PMC6416337; doi:10.1038/s41467-019-09181-2)
Supplement: Supplementary file 3 — Description of Additional Supplementary Files [file 41467_2019_9181_MOESM3_ESM.docx]

**Description of Additional Supplementary Files**

File Name: Supplementary Data 1

Description: MS/MS data
